# Emergency temporary standards and COVID-19 trends among Oregon farmworkers

**DOI:** 10.1371/journal.pone.0329130

**Published:** 2025-08-08

**Authors:** Raul Cruz-Cano, Devon C. Payne-Sturges

**Affiliations:** 1 Department of Epidemiology & Biostatistics, School of Public Health, Indiana University, Bloomington, Indiana, United States of America; 2 Department of Global, Environmental, and Occupational Health, University of Maryland School of Public Health, College Park, Maryland, United States of America; Hamadan University of Medical Sciences, IRAN, ISLAMIC REPUBLIC OF

## Abstract

**Background:**

During the COVID-19 pandemic, migrant and seasonal farmworkers were deemed essential due to their central roles in US agricultural operations. However, employer-provided housing and transportation conditions increased their risks of SARS-CoV-2 exposure, and some states implemented emergency temporary standards (ETSs) at the insistence of farmworker advocates. Despite numerous studies examining the effectiveness of policy interventions (e.g., workplace closures) for mitigating SARS-CoV-2 transmission, limited research has specifically examined the effectiveness of interventions aimed at protecting farmworkers from COVID-19.

**Methods:**

We used an interrupted time series analysis to estimate how two ETSs and one executive order issued in Oregon impacted COVID-19 trends from March 1, 2020, to February 27, 2021, for the overall population and among agricultural labor groups in Oregon.

**Results:**

Our models show that the ETS and executive order, which specifically targeted farmworker housing, transportation, and worksites, did not demonstrate any significant effects on the numbers of COVID-19 cases or associated deaths. However, the other ETS, which targeted all workplaces, was associated with statistically significant decreases in COVID-19 cases among the general population (−142.36214, p-value<0.0001), producers (−1.67128, p-value = 0.0009), hired workers (−2.39413, p-value = 0.0014), unpaid workers (−1.01572, p-value = 0.0003), and migrant workers (−0.60017, p-value = 0.0166). None of the three policy changes were found to have any statistically significant impacts on the numbers of COVID-19–associated deaths.

**Conclusions:**

The ETS targeting all workplaces was more effective for reducing COVID-19 transmission than the ETS or executive order specifically targeting farmworkers, indicating that the design, communication, and implementation of ETSs targeting farmworkers should be re-evaluated.

## Introduction

During the COVID-19 pandemic, Black and Latino/a migrant and seasonal farmworkers (MSFWs) were deemed essential workers due to the central roles they play in U.S. agricultural operations [1]. In addition to the dangers and inequities that farmworkers already face [2–6], these workers often live in rural and isolated areas [4,7,8] and are subjected to employment-related working, housing, and transportation conditions that increase their risks of exposure to highly contagious respiratory viruses, such as SARS-CoV-2 [9–15]. During the early months of the pandemic, numerous COVID-19 outbreaks were reported among hired farmworkers and at farm labor camps [16–21]. Epidemiological studies showed that farmworkers experienced an increased risk of developing COVID-19 [22–27], and researchers at Purdue University estimated that over 353,000 MSWFs were infected with SARS-CoV-2 [28], Some state governments implemented binding emergency temporary standards (ETS) [29], but at the height of the pandemic, the federal government had issued only nonbinding guidance [30]. Although a belated federal ETS was eventually issued, it was limited to health care workers [31,32], leading labor, public health, and farmworker advocates to demand increased implementation of protective measures at the federal level [33–36].

Previous studies have attempted to evaluate whether state-level policy interventions and governor-issued executive orders are effective in mitigating SARS-CoV2 transmission among the general public; examples of such policies include attempts to increase physical distancing (e.g., limiting group sizes, closing public schools and non-essential businesses, canceling public events, lockdown orders) and mask mandates [37–44]. However, few, if any, of these studies have accounted for the high correlation between daily COVID-19 caseloads and observed or estimated values for the previous day (secular trend) or week (weekly cycle), which has the potential to result in the true effects of interventions being either overestimated or underestimated [45–48]. In addition, limited research has focused on the comparative effectiveness of interventions specifically aimed at protecting MSFWs from COVID-19 [49] while accounting for the well-documented secular trends and weekly cycles of COVID-19 infections [50–52]. Given the structural inequities often experienced by MSFWs [10,53–56], health policy interventions may not offer the level of health protection reported for other occupational settings, and differences across demographic groups may occur [57]*.* Thus, understanding the differential impacts of occupational and public health policies aimed at preventing COVID-19 transmission remains an important knowledge gap, particularly as such differences may represent barriers to the effectiveness of occupational health policies.

We conducted a natural experimental study using an interrupted time series (ITS) analysis with autoregressive integrated moving average (ARIMA) models to examine COVID-19 caseloads and mortality trends in the state of Oregon before and after the implementation of three statewide policies aimed at protecting agricultural workers, including MSFWs, during the first year of the COVID-19 pandemic (March 1, 2020, to February 27, 2021). ITS analyses are useful for examining the impacts of an intervention because they maximize the analytic benefits of a large number of repeated observations over a period of time [58,59] and, when combined with ARIMA, can account for expected secular and weekly trends in outcomes of interest [60]. The ITS approach has been successfully applied to evaluating the impacts of various COVID-19 mitigation policies among the general public [37–40,61] but has not yet been applied to assessing the impacts of mitigation policies among agricultural workers while using ARIMA models. We hypothesize that each intervention (two ETSs and one executive order) would be associated with reductions in in COVID-19 cases and deaths after a delay accounting for incubation, with differential effects by agricultural worker types given the structural inequalities hired and migrant farmworkers face in terms of living in poor quality congregate housing [7,14], exclusion from sick leave policies [10], and lack of access to health care [9,62,63]. Additionally, we assumed an abrupt impact of these policies on cases but a gradual impact on deaths.

## Methods

### Study design

We designed our study to assess changes in COVID-19 trends among agricultural workers before and after the issuance of two ETSs and one executive order in Oregon, intended to control, prevent, and mitigate SARS-CoV-2 transmission among employees and employers. On May 11, 2020, Oregon Occupational Health and Safety Administration (Oregon OSHA) issued the first ETS, effective immediately and remaining in effect until repealed, but no later than October 25, 2020, whichever occurred first; this ETS addressed COVID-19 in employer-provided housing, labor-intensive agricultural operations, field sanitation, and transportation [64]. Employers were required to institute social distancing measures (maintaining 6-ft distance) and increase the availability of toilet and handwashing facilities from one for every 20 workers to one for every 10 workers [64]. Additionally, bunk bed use by unrelated people was prohibited, and employers were required to separate beds by at least 6 feet of space or install an impermeable barrier [64]. When transporting multiple farmworkers, facial coverings, social distancing (at least 3-ft), and sanitation of the vehicles were required (all high contact surfaces must be sanitized before each trip or at least 2 times daily) [64]. Other requirements included the isolation of individuals with suspected or confirmed COVID cases in separate sleeping, eating and bathroom accommodations away from other who are not sick [64].

Faced with the continuing threat of rapid COVID-19 transmission in settings where workers live in crowded housing conditions and work in close quarters, even during the agricultural off-season, the Governor of Oregon issued Executive Order 20–58, which extended Section 2 of the first ETS, primarily addressing employer-provided housing, including social distancing measures, toilet to worker ratio, and bed regulations [65]. This executive order was effective from October 23, 2020, to April 30, 2021 [65].

Oregon OSHA issued a second ETS, effective November 16, 2020 through May 4, 2021, that applied to workplaces within the Oregon OSHA jurisdiction, including farmworkers and their employers [66]. Employers were required to enact a comprehensive set of risk-reducing measures, including physical distancing, the use of mask and face coverings, improving ventilation, conducting exposure risk assessments, developing an infection control plan, providing training on these measures, notifying workers of workplace COVID-19 infections and possible exposures to infected persons, COVID-19 testing, and compliance with “medical removal” of an employee if recommended by the Oregon Health Authority, local public health agency or medical provider due to quarantine or isolation for COVID-19. At the time, this ETS was the only state standard that specifically required employers to obtain employee suggestions and feedback on COVID-19 mitigation plans before implementation. Although the hierarchy of controls [67], an important occupational health paradigm, is not explicitly discussed in this ETS, the need for a layered approach for controlling exposures to “COVID-19 hazards” is specified [66].

Our population-based ITS analysis focuses on COVID-19 trends in Oregon between March 1, 2020, and February 27, 2021, during the first year of the COVID-19 pandemic. Detailed comparisons of each policy intervention are presented in supporting information (S1Table and S2 Table). S1 Fig provides an overview of the study timeline. This period allowed for several weeks of observation after the first ETS was issued. This study used publicly available data and was approved by the institutional review board at the University of Maryland.

### Data sources

The tracking of COVID-19 trends among farmworkers has been hampered by a lack of routine data collection and reporting on COVID-19 cases by occupation at both the state and federal levels. Researchers at Purdue University published a methodology for estimating the number of COVID-19 cases and deaths among agricultural workers across US counties and over time [68]. Briefly, Lusk et al. assume that in any given county, agricultural workers contract COVID-19 at a rate equal to that of the general population [68]. Thus, the expected number of agricultural workers who have contracted or died from COVID-19 can be calculated by multiplying the proportion of the total population represented by agricultural workers by the total number of COVID-19 cases or deaths. Lusk et al. applied their approach using county-level COVID-19 data from the Johns Hopkins University COVID tracker [68]. The Census of Agriculture, conducted by the National Agricultural Statistics Service, is considered the “only source of uniform, comprehensive and impartial agricultural data for every county in the United States” [68]. The Census of Agriculture provides estimates of the numbers of agricultural workers by type: (1) **agricultural producer** (often interpreted as “farmer” or “farm owner”); (2) **hired farm** or ranch workers (including paid family members and migrant workers not paid on contract); (3) **unpaid farm** or ranch workers (including unpaid family members of the “producer”); and (4) **agricultural migrant workers** (both hired and contract labor) [68,69]. As noted by Lusk et al., these categories are not mutually exclusive and should not be summed [68].

We extended and improved upon the methodology by Lusk et al. by utilizing the most current Census of Agriculture (data accessed September 3, 2024) [69] and interpolating data on agricultural workers from two US Department of Agriculture censuses (2017 and 2022) to more accurately estimate the numbers of agricultural workers during 2020 and 2021 (see S1 Text for more details). Using population estimates for each Oregon county from the US Census Bureau for the years 2020 and 2021 [70], we applied the Lusk et al. apportionment methodology to Oregon COVID-19 case and mortality data (based on tracking data from Johns Hopkins University [71] from March 1, 2020 to February 27, 2021.

### Independent variables

The primary independent variables were the effective dates of the two Oregon ETSs and the executive order, which we obtained from the Oregon Secretary of State’s website [64,65,72]. Because ITS analysis requires a clear differentiation between the pre-intervention period and the post-intervention period, each policy was encoded as a separate binary variable, equal to 0 before the effective date and 1 on the effective date and thereafter [73]. The first binary variable, *ETS1-housing*, combined the ETS regulating agricultural labor housing requirements and the executive order mandating the continuation of those requirements; *ETS1-housing* was set to 0 from March 1, 2020, to May 10, 2020, and to 1 from May 11, 2020, to February 27, 2021. The second binary variable, *ETS1-transport*, represented all transportation and field sanitation requirements from the first ETS; *ETS1-transport* was set to 0 from March 1, 2020, to May 10, 2020; to 1 from May 11, 2020, to October 23, 2020; and to 0 from October 24, 2020, to February 27, 2021. The third binary variable, *ETS2*, represented the ETS for all workplaces; *ETS2* was set to 0 from March 1, 2020, to November 15, 2020, and to 1 from November 16, 2020, to February 27, 2021.

### Outcome measures

The primary outcomes or dependent variables were the 7-day rolling mean of daily COVID-19 cases and deaths at the county level in Oregon from March 1, 2020, to February 27, 2021. We used the 7-day rolling mean to be consistent with similar studies [44]. Data on COVID-19 cases and deaths were from Johns Hopkins University, made available by Lusk et al. at their data repository [71]. As described earlier, population data from the US Census Bureau for the years 2020 and 2021 [70] were used to calculate rates per 100,000 population.

### Statistical analysis

We used the Box–Jenkins approach [74] to build a set of adequate seasonal ARIMA (SARIMA) models to represent both secular and weekly COVID-19 trends. We estimated the SARIMA model parameters using the maximum likelihood method (e.g., Luz et al. [75]). We used the Akaike information criterion to select the most appropriate model and ensure the accuracy of the model for capturing outcome variable behaviors before the intervention was implemented (see S2 Text). All preliminary experiments were conducted in JMP 17 Pro [76].

Unlike a simple segmented ITS regression, which assumes a linear time series, SARIMA models are designed to capture complex temporal structures such as autocorrelation and weekly seasonality, both of which are prominent features in COVID-19 case and death data. Moreover, segmented regression is not always adequate when autocorrelation or seasonality is present, and ARIMA models provide a valid alternative in such cases [73]. Our study includes a year of daily observations from March 1, 2020, to February 27, 2021, exceeding the 50-observation threshold recommended for ARIMA models [74] and allowing robust estimation of both short- and medium-term effects. The intervention analysis in the SARIMA model is not restricted to modeling changes in level and slope but can also be used to assess complex patterns that occur following intervention implementation, such as abrupt impacts, gradual shifts, or pulses that slowly diminish over time, while also accounting for lagged effects [60] tied to incubation periods and reporting delays. This method has been successfully applied in recent public health policy evaluations, including [77], who used ARIMA to assess COVID-19 policy impacts across China. Transfer functions are used to model more sophisticated relationships between an intervention and the outcome series, *Y*_*t*_ [78–80].

As mentioned previously, we analyzed daily counts of COVID-19 cases and deaths from Johns Hopkins University and used interpolated population estimates derived from the USDA Census of Agriculture and U.S. Census Bureau to calculate agricultural worker-specific outcome rates.

The binary intervention variables, $X_{t}$, are defined as a pulse function:


$$X_{t}=\{0\;for\;t=T,\;1\;for\;t\neq T\}$$ (1)

where the time period *T* is defined by the days for which the variable is set to 1. The decision to include only the explanatory variable of interest in a time series intervention model is driven by the need to accurately capture and interpret the effects of a specific intervention while maintaining simplicity and statistical validity. All other variables that might influence the outcomes are thought to be “baked into” the time series. In a time series model, a variable serves as its own control because the past values of the variable are incorporated as predictors, leveraging the autoregressive nature of the data to capture the influence of the variable’s history on its current behavior.

When modeling COVID-19 cases, we treated the Oregon policies as having an abrupt, permanent impact on them. Delavary et al. [81] and Humphreys et al. [80] show that this can be represented as:


$$\begin{array}{c} Y_{t}=\mu +\sum {\mathrm{\backslash nolimits}}_{i=1}^{3}\left(\;\omega_{i}B^{h}\right)X_{i,t}+N_{t} \end{array}$$ (2)

where *h* represents the lag of the effect [73] and ***B*** is the backshift operator (i.e., ***B***^***p***^ = *Y*_*t − p*_). The effects of each policy implementation are expected to be delayed by ***p*** days, which reflects the difference in time that exists between infection and case reporting due to, among other causes, COVID-19 incubation period, the outcome reporting time, and the time required for policy implementation. Therefore, we lagged the outcomes based on cross-correlations and best-fit parameters from the SARIMA model. The index, *i,* tracks the law represented by the transfer function. A negative$\;\omega_{i}$ value would represent a drop in the outcome variable, $Y_{t}$. $X_{i,t}$ represents the intervention variable, and $N_{t}$ represents the SARIMA model identified earlier.

A model with a gradual, permanent effect can be represented as:


$$\begin{array}{c} Y_{t}=\mu +\sum {\mathrm{\backslash nolimits}}_{i=1}^{3}\left(\;\omega_{i}{/(1-\delta_{i}B}^{h})\right)X_{i,t}+N_{t}\; \end{array}$$ (3)

A negative scale parameter,$\;\omega_{i}$, would represent a drop in $Y_{t}$ due to law *i*, whereas the denominator parameter $\delta_{i}$ would indicate the size of the change.

We selected different transfer functions for the analyses of cases and deaths. The transfer function models the effect of the ETS, not the ETS itself (which is the intervention variable). Regardless of whether the effect is assumed to be gradual or abrupt, the ETS itself is represented in the dataset as a binary variable because we assumed an immediate implementation. However, an abruptly implemented ETS may not have an immediate effect on COVID-19 deaths. Policy interventions do not affect the pathological condition of individuals who have already been infected or are hospitalized, and these individuals may die after the intervention has been implemented, resulting in a delayed stabilization of the death rate under the new policy. This delay is analogous to pressing the brake pedal in a car: stepping on the brake pedal abruptly does not stop the car instantly, as inertia causes the car to continue moving.

When considering COVID-19 cases, the ‘pipeline’ might be short enough to overlook. For example, Faes et al. [82] showed that the interquartile range for the time from infection to death is 7–37 days, with hospitalization lasting 4 to 7 days. The time from symptom onset to hospitalization ranges from 1 to 10 days, whereas the time from hospitalization to death ranges from 2 to 20 days. Although a change in case numbers over a few days period can be considered abrupt, a change in deaths over a month-long period should be considered gradual.

All statistical analyses were performed using SAS statistical software (version 9.4), and 2-sided p-values less than 0.05 were considered statistically significant.

## Results

Table 1 shows the estimated number of COVID-19 cases and deaths among four types of agricultural workers in Oregon from March 1, 2020, until February 27, 2021. We estimated 2,752 cases among producers, 3,862 cases among hired workers, 1,767 cases among unpaid workers, and 962 cases among migrant workers. Migrant workers had the highest estimated case incidence rate, at 4,557 cases per 100,000 migrant worker population, and the highest estimated death rate, at 81 deaths per 100,000 migrant worker population.

**Table 1:** Estimated number of COVID-19 cases and deaths among various types of agricultural workers in Oregon from March 1, 2020, to February 27, 2021.

Type of Worker	Total Population	Total Cases	Case rate per 100,000	Total Deaths	Death Rate per 100,000
Producers	68,773	2,752.48	4,002.27	40.63	59.08
Hired Workers	86,240	3,862.79	4,479.12	62.11	72.02
Unpaid Workers	45,713	1,766.92	3,865.25	26.05	56.98
Migrant Workers	21,131	962.86	4,556.61	17.11	80.98
Total Oregon Population	8,287,250	154,457.40	1,863.80	2,191.14	26.44

Note: Cases, deaths, and population counts are averages based on the interpolated population numbers: (1) **agricultural producer** (often interpreted as “farmer” or “farm owner”); (2) **hired farm** or ranch workers (including paid family members and migrant workers not paid on contract); (3) **unpaid farm** or ranch workers (including unpaid family members of the “producer”); and (4) **agricultural migrant workers** (both hired and contract labor).

**Fig 1:**
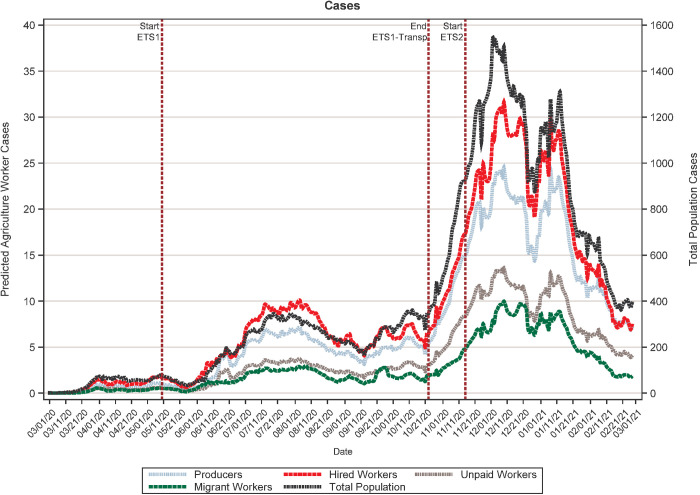
Trends in COVID-19 cases in Oregon, for both the overall population and by agricultural worker type, between March 1, 2020 and February 27, 2021.

**Fig 2:**
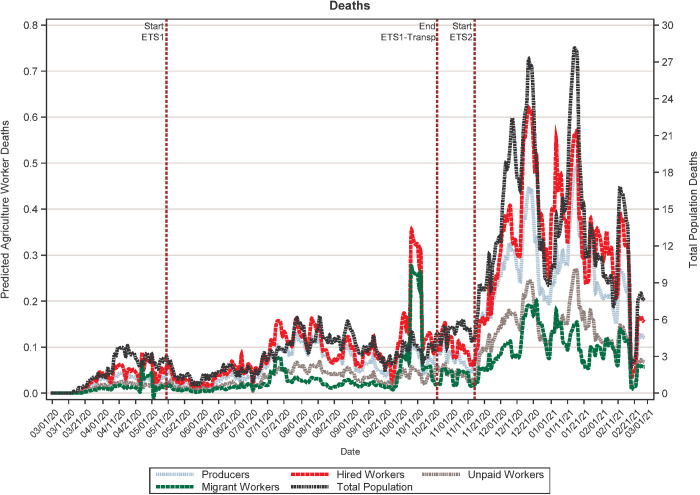
Trends in COVID-19 deaths in Oregon, for both the overall population and by agricultural worker type, between March 1, 2020 and February 27, 2021.

Figs 1 and 2 presents the 7-day rolling average of daily COVID-19 cases and deaths in Oregon from March 1, 2020, until February 27, 2021, for the four agricultural labor categories and the general population. Prior to the implementation of the housing requirements in the first ETS, the average rates of new COVID-19 cases and deaths were increasing among farmworkers. Trends in the number of cases among producers and unpaid workers closely mirrored those for the general population (Table 2), indicating that the number of cases among farm owners/operators (correlation coefficient: 0.901) and their family members (correlation coefficient: 0.905) increased as cases increased among the general population. Although still positive, the correlations between trends in COVID-19 cases among migrant workers (correlation coefficient 0.886) and hired workers (correlation coefficient: 0.775) and those for the general population were weaker than for producers and unpaid workers, suggesting different COVID-19 transmission dynamics.

**Table 2:** Correlation between the 7-day rolling average of estimated new daily COVID-19 cases among agricultural labor groups and the general population in Oregon.

Statistic	All Ag. Workers	Producers	Unpaid	Hired	Migrant
Mean	0.901	0.901	0.905	0.886	0.775
Median	0.926	0.928	0.935	0.919	0.857
IQR	0.118	0.125	0.110	0.114	0.220
Minimum	0.0180	0.1721	0.151	−0.311	−0.430

Note:Ag., agricultural; IQR, interquartile range.: (1) **agricultural producer** (often interpreted as “farmer” or “farm owner”); (2) **hired farm** or ranch workers (including paid family members and migrant workers not paid on contract); (3) **unpaid farm** or ranch workers (including unpaid family members of the “producer”); and (4) **agricultural migrant workers** (both hired and contract labor).

Our models show that neither ETS1-housing, which included the ETS addressing agricultural labor housing and the executive order extending these requirements, nor ETS1-transport had an impact on trends in new COVID-19 cases (Table 3). However, ETS2, targeting all Oregon workplaces, was associated with statistically significant decreases in COVID-19 cases for the general population (−142.36214, p-value<0.0001), producers (−1.67128, p-value = 0.0009), hired workers (−2.39413, p-value = 0.0014), unpaid workers (−1.01572, p-value = 0.0003) and migrant workers (−0.60017, p-value = 0.0166). No statistically significant changes in deaths (Table 4) were detected for any of the three variables included in this study.

**Table 3:** Estimated coefficients from the SARIMA model of new COVID-19 cases, assuming an abrupt effect following the implementation of two ETSs and one executive order in Oregon.

SARIMA Model: (1,1,0)x(1,0,1)_7_		General Population		Producers		Hired Workers		Unpaid Workers		Migrant Workers	
Effect	Param.	Value	p-value	Value	p-value	Value	p-value	Value	p-value	Value	p-value
	μ	1.56432	0.3024	0.04815	<.0001	0.02877	0.3923	0.02614	<.0001	0.0076479	0.4611
		0.46845	0.0129	0.99998	<.0001	0.37064	0.0387	0.99998	<.0001	0.45553	0.0008
		0.14553	0.0121	0.1568	0.0048	0.12987	0.0218	0.16633	0.0027	0.14724	0.0106
		0.235	0.2491	0.78138	<.0001	0.09119	0.6287	0.79361	<.0001	0.09335	0.5224
ETS1-Housing	ω_1_	9.79788	0.8343	0.1931	0.781	0.18671	0.8595	0.11042	0.7738	0.04007	0.9096
ETS1-Transport	ω_2_	−16.74797	0.6127	−0.26158	0.5811	−0.40533	0.5864	−0.17028	0.5176	−0.10747	0.6662
ETS2	ω_3_	−142.36214	<.0001	−1.67128	0.0009	−2.39413	0.0014	−1.01572	0.0003	−0.60017	0.0166

Note: ETS1- Housing is; ETS1-Transport is; ETS2 is. 1) **agricultural producer** (often interpreted as “farmer” or “farm owner”); (2) **hired farm** or ranch workers (including paid family members and migrant workers not paid on contract); (3) **unpaid farm** or ranch workers (including unpaid family members of the “producer”); and (4) **agricultural migrant workers** (both hired and contract labor).

**Table 4:** Estimated coefficients from the SARIMA model of new COVID-19 deaths assuming a gradual effect following the implementation of two ETSs and one executive order in Oregon.

SARIMA Model: (1,1,0)(0,0,1)_7_		General Population		Producers		Hired Workers		Unpaid Workers		Migrant Workers	
Effect	Param.	Value	p-value	Value	p-value	Value	p-value	Value	p-value	Value	p-value
	μ	0.02047	0.6277	0.000324	0.6406	0.000433	0.6316	0.000179	0.6287	0.00022	0.0529
		0.48481	<.0001	0.43443	<.0001	0.51156	<.0001	0.44219	<.0001	0.99997	<.0001
		0.32775	<.0001	0.09557	0.0888	−0.00395	0.9448	0.11941	0.0325	0.02091	0.7155
ETS1-Housing scale	ω_1_	0.4752	0.5971	0.01052	0.5943	0.01168	0.7074	0.005494	0.5986	0.006234	0.4788
ETS1-Housing denominator	δ _1_	−0.18466	0.915	0.07929	0.9651	0.0852	0.9735	0.07643	0.9665	0.85555	0.0095*
ETS1-Transport scale	ω_2_	−0.0048	0.9903	−0.00031	0.9731	0.000593	0.9672	−3.9E-05	0.9936	−0.00477	0.3774
ETS1-Housing denominator	δ _2_	0.99993	0.9514	0.99997	0.8619	1	0.8303	−0.99963	0.9673	0.22839	0.8613
ETS2 scale	ω_3_	−0.12565	0.8778	−0.0035	0.8334	−0.00366	0.8909	−0.0026	0.7692	−0.00882	0.2238
ETS2 denominator	δ _3_	−0.37477	0.944	−0.64828	0.8275	−0.58597	0.9037	−0.70028	0.728	−0.59029	0.2803

Note: ETS1- Housing is; ETS1-Transport is; ETS2 is. 1) **agricultural producer** (often interpreted as “farmer” or “farm owner”); (2) **hired farm** or ranch workers (including paid family members and migrant workers not paid on contract); (3) **unpaid farm** or ranch workers (including unpaid family members of the “producer”); and (4) **agricultural migrant workers** (both hired and contract labor).

## Discussion

The results of the present study show the varying impacts of two ETSs and one executive order in Oregon on trends in COVID-19 cases and deaths among agricultural workers from March 1, 2020, to February 27, 2021. Of the three policies examined, the ETS applied to all workplaces (*ETS2*) appeared to have the most positive impact and was statistically significantly associated with decreases in COVID-19 cases for the general population and all agricultural labor categories in Oregon. To the best of our knowledge, this is one of the first studies to evaluate the impacts of different ETSs on COVID-19 trends among farmworkers using ITS analysis with SARIMA models.

Oregon, one of 22 states with a federally approved state OSHA program, has authority to tailor its workplace safety regulations to meet or exceed Federal OSHA requirements [55], such as through the issuance of ETSs to protect against immediate threats to worker safety and health [55]. ETSs are effective upon publication and remain valid for up to 6 months, allowing time for procedural requirements and permanent standards [55]. During the early months of the COVID-19 pandemic, large outbreaks were reported at meat processing facilities and among workers on fruit and vegetable farms, including in Oregon [83,84], motivating worker safety groups to push for state-level ETSs due to a lack of action at the federal level. Pursuing policy interventions through state-level ETSs increases the likelihood of success due to existing implementation mechanisms.

Oregon issued its first ETS on May 11, 2020, aiming to reduce the risk of COVID-19 transmission among farmworkers by mandating 6-foot physical distancing at work sites and in labor housing; allowing farm operators to erect plywood or plexiglass barriers between beds if 6-foot separation was not possible; mandating increased access to toilet and handwashing facilities (1 for every 10 workers rather than the existing federal and state standard of 1 for every 20 workers); requiring that transportation vehicle surfaces be cleaned; and requiring that cloth facial coverings be worn during transport. Although training workers on how to minimize COVID-19 transmission was not included in this ETS, employers were required to post information about good hygiene practices at worksites. However, our study showed that *ETS1-housing*, which included the housing requirements in the first ETS and their extension via the executive order, had no measurable effect on reducing COVID-19 cases or deaths, which is not surprising, as farmworker advocacy organizations noted a number of potential deficiencies at the time of this ETS issuance [85]. For example, the recommendation of 6-foot physical distancing by the Centers for Disease Control and Prevention was intended to prevent transmission during brief, incidental encounters [86,87], and was likely insufficient for protecting farmworkers who reside in congregate housing. In addition, the first ETS did not require improvements in ventilation at agricultural labor housing locations, reductions in occupant density in sleeping quarters, or routine COVID-19 testing to monitor asymptomatic cases. A survey of Oregon farmworkers several months after the issuance of the first ETS revealed that although employers were taking some steps to comply with the ETS, workers were not always provided with proper protection gear, were frequently unable to maintain 6-foot physical distancing while working, and were not able to quarantine or isolate when infected [88]. A review of violations data from Oregon OSHA by authors of the farmworker survey suggested that agricultural inspections, including inspections of employer-provided housing, declined during 2019–2021 [89], indicating ineffective implementation.

By contrast, the second ETS, which targeted all Oregon workplaces, was statistically significantly associated with decreases in new COVID-19 cases for all agricultural labor groups but had no impact on COVID-19 deaths. This ETS incorporated more comprehensive control strategies, including screening, ventilation, contact tracing, and medical removal procedures. This ETS also incorporated the concept of a hierarchy of controls [67], an important occupational health paradigm that prioritizes strategies intended to eliminate or reduce the presence of hazards in the workplace over those that require changes in worker behaviors or the use of personal protective equipment. By the time the second ETS was issued, more was known about the virus, and protective behavioral changes were already being practiced. In addition, Oregon OSHA announced its intention to issue the second ETS months in advance and solicited public input.

Previous studies have demonstrated that non-pharmacological strategies have had substantial effects on reducing COVID-19 trends among the general public [37–40]. However, these studies examined shorter periods (e.g. Feb – May 2020) than our study, which examined the entire first year of the pandemic, including the fall and winter months, during which COVID-19 transmissions and deaths increased dramatically. The increase in COVID-19 transmission during the fall and winter months may explain why none of the examined policies were associated with decreases in the daily COVID-19 mortality rate. Note, *ETS1-housing* remained in effect until April 30, 2021 and *ETS2* went into effect November 16, 2020 (see SI Fig 1 for overview of timeline). Additionally, the rural communities in which Oregon’s agricultural workers are located may have experienced higher mortality rates as compared to non-rural areas early in the pandemic, as older and rural residents experience greater risks of serious illness and mortality due to COVID-19 [90].

Our study has several strengths. We employed a robust methodological and analytical approach to evaluate the impacts of state-level policies on COVID-19 cases and deaths. We used SARIMA models, which are designed to handle autocorrelation, seasonal trends, and other regular variations in data. Unlike a straightforward segmented ITS regression, which are based on linear models, ARIMA models more effectively capture temporal structures and are not limited to examining changes in level or slope but can evaluate more intricate patterns related to the intervention. Our models were built using data from the 2022 Census of Agriculture and interpolated population estimates to more closely align with the study period. Our study period is much longer than most earlier COVID-19 ITS studies, in which the short observation times were noted as limitations. Another strength of this study is that we modeled the cumulative impact of multiple policies across four categories of farm laborers, as defined by the Census of Agriculture. Our study results show that the comprehensive implementation of workplace COVID-19 exposure risk control strategies successfully reduced the incidence of COVID-19 cases across all agricultural worker categories but did not reduce deaths.

Our study also has some limitations. Similar to other COVID-19 studies, we relied on publicly available tracking platforms to obtain daily case and death data, and these may be impacted by measurement error, such as the underrepresentation of asymptomatic cases and out-of-hospital deaths. Changes in the definitions [91] of COVID-19 cases and deaths during the study period (e.g., starting April 14, 2020, aggregate and individual counts included confirmed and probable cases and deaths, according to the Council of State and Territorial Epidemiologists position statement Interim 20-ID-01 [90]) could also our models’ ability to detect a relationship between the outcomes and the policy interventions. Reporting inconsistencies are unavoidable limitations of all COVID-19 population-based studies; however, recent evaluations show the high reliability of our data source [92]. The data in the Census of Agriculture is not perfect, and some agricultural worker categories overlap. Following the Lusk et al. approach, we assumed that the numbers of COVID-19 cases and deaths among agricultural workers in each county were proportional to their population share, which may not be accurate. Population changes across worker categories may not follow linear trends. Although we tested several SARIMA models, an unexplored model could have provided a better fit. Variations in the transfer function and lag size can also lead to different results. Our analysis cannot address policy implementation or compliance; however, we drew upon media reports and surveys for this context. While our study shares common limitations with many observational studies conducted during the COVID-19 pandemic, such as potential underreporting, overlapping worker classifications, and data inconsistencies, we note that these sources of bias likely remained stable over the 12-month study period. Since our goal was not to forecast outcomes but to detect changes in trends following policy implementation, stable biases would not invalidate the internal comparisons made within the time series. Additionally, although we did not exhaustively test all possible SARIMA or transfer function configurations, we relied on established Box-Jenkins procedures and diagnostic tools (e.g., ACF, PACF, IACF) to select well-fitting models. As such, we believe our findings offer a robust and methodologically sound assessment of the intervention effects despite the inherent data challenges.

## Conclusion and implications

The COVID-19 pandemic highlighted enduring racial and social inequities in occupational health, as minoritized workers were forced to remain on the job and risk exposure to SARS-CoV-2 to ensure that the US economy remained functional, resulting in a higher risk of COVID-19 infection among farmworkers and meat processors [22–27]. Designating agricultural workers as essential at the start of the COVID-19 pandemic increased their risks of contracting COVID-19. This increased risk is not an anomaly, as the agricultural labor system in the US routinely places hired workers in dangerous worksite and housing conditions. However, the implementation and enforcement of occupational health and safety standards can mitigate the impacts of workplace hazards. Although the U.S. federal government did not take action, some state governments issued ETSs and executive orders intended to protect farmworkers. However, limited systematic evaluations have examined the effectiveness of interventions specifically aimed at protecting MSFWs from COVID-19. Drawing upon existing studies evaluating the impacts of policy interventions on preventing COVID-19 transmission among the general public, we conducted a quasi-experimental study to examine the impacts of state-level policies aimed at protecting agricultural workers from COVID-19 in Oregon. We used ITS analysis and SARIMA models, and our results showed that two ETSs and one executive order had varying impacts on COVID-19 trends among agricultural workers between March 1, 2020, and February 27, 2021. The ETS that applied to all workplaces appeared to have had a more positive impact than the ETS or executive order that applied only to agricultural workers. The all-workplace ETS was statistically significantly associated with decreases in COVID-19 cases among the general population and across all agricultural labor categories, suggesting that the implementation of comprehensive workplace COVID-19 exposure risk control strategies can reduce the incidence of cases; however, no impact on deaths was observed for any policy.

Our study demonstrates the application of ITS analysis to assess effectiveness of occupational health and safety policy interventions, contributing to ongoing policy discussions regarding how best to prevent the workplace transmission of respiratory infections among hired farmworkers. Future research should evaluate regulatory interventions across multiple states to understand which infection control strategies are the most effective. Ideal studies would combine ITS analysis with qualitative data on intervention implementation (e.g., surveys of farmworkers, employers, and governmental agency staff).

## Supporting information

S1 Fig Overview of Timeline of Study Period. (DOCX)

S1 Table Oregon ETSs and Governor’s Executive Order Effective Dates, Scope, Definitions of Hazard and Exposure, and Risk Assessment Requirements. (DOCX)

S2 Table Control Strategies Required by each Oregon ETS and Governor’s Executive Order. (DOCX)

S1 Text Interpolating general population and number of agricultural in Oregon. (DOCX)

S2 Text Seasonal ARIMA model selection. (DOCX)
