# Supplementary figures and images for: Emergency temporary standards and COVID-19 trends among Oregon farmworkers
Source: PLoS One. 2025 Aug 8;20(8):e0329130. doi: 10.1371/journal.pone.0329130 (PMC12334050; doi:10.1371/journal.pone.0329130)

S1 Figure. Overview of Timeline of Study Period

**
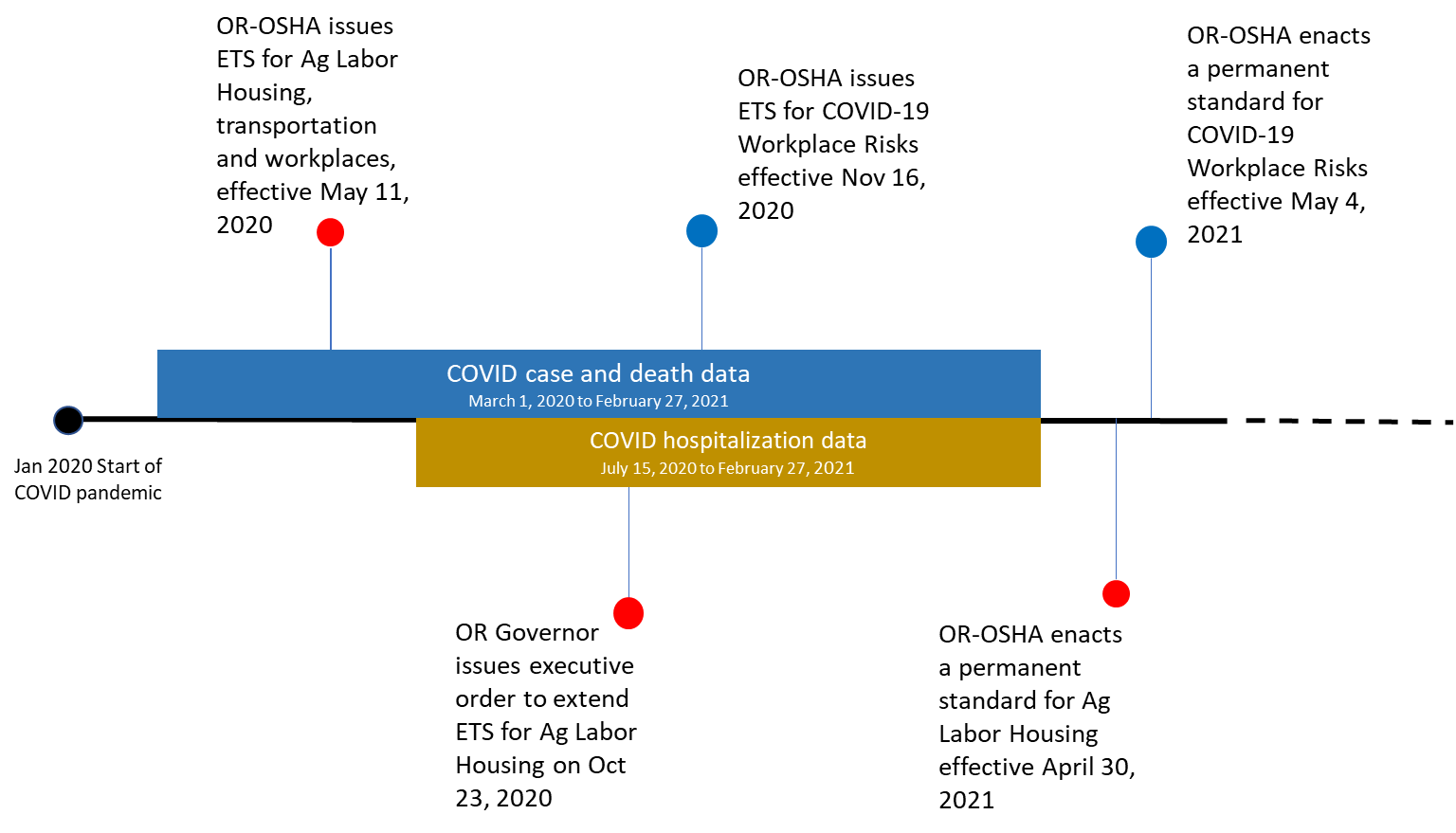
**

Supplement: S1 Fig — (DOCX) [file pone.0329130.s001.docx]
