# Supplementary material for: Emergency temporary standards and COVID-19 trends among Oregon farmworkers
Source: PLoS One. 2025 Aug 8;20(8):e0329130. doi: 10.1371/journal.pone.0329130 (PMC12334050; doi:10.1371/journal.pone.0329130)
Supplement: S1 Table — (DOCX) [file pone.0329130.s002.docx]

S1 Table. Oregon ETSs and Governor’s Executive Order Effective Dates, Scope, Definitions of Hazard and Exposure, and Risk Assessment Requirements

| Title | Effective Date | Scope | Hazard | Exposure(s) | Risk Assessment Required | Basis of Risk Determination | Risk Categories |
| --- | --- | --- | --- | --- | --- | --- | --- |
| **Temporary rule Addressing the COVID-19 Emergency in Employer-Provided Housing, Labor-Intensive agricultural Operations , and Agricultural Transportation OAR 437-001-0749** (ETS1- transport) | 5/11/2020 | All labor-intensive agricultural operations; all temporary worker housing or other employer-provided housing (but not hotels/motels that provide similar housing commercially to the  public); and all employer-provided transportation within labor-intensive agricultural operations | COVID-19, suspected and confirmed cases | Contact, contaminated  surfaces | No | Not discussed | Not discussed |
| **Executive Order 20-58 extending employer-provided housing requirements (Section 2) of OAR 437-001-0749** (ETS1-housing) | 10/23/2020 | All temporary  worker housing or other employer-provided housing (but not hotels/motels that provide similar housing commercially to the  public) | COVID-19, suspected and confirmed cases | Contact, contaminated  surfaces | No | Not discussed | Not discussed |
| **Temporary Rules Addressing the COVID-19 Public Health Emergency in All Oregon Workplaces OAR 437-001-0744** (ETS2) | 11/16/2020 | Employees, all employers with one or more employees, and places of employment under jurisdiction of OR OSHA | COVID-19, individual who has tested positive for COVID-19* | Droplets, contact, airborne modes | Yes | Per definition in Section 1 to determine if exceptional risk, otherwise general workplace | Workplaces at exceptional risk; all other workplaces |
