# Supplementary material for: Emergency temporary standards and COVID-19 trends among Oregon farmworkers
Source: PLoS One. 2025 Aug 8;20(8):e0329130. doi: 10.1371/journal.pone.0329130 (PMC12334050; doi:10.1371/journal.pone.0329130)
Supplement: S2 Table — (DOCX) [file pone.0329130.s003.docx]

S2 Table. Control Strategies Required by each Oregon ETS and Governor’s Executive Order

|  | **ETS1- farm labor housing, work sites and transport** | **ETS1 – farm labor housing only (Governor’s Exec Order)** | **ETS2 – all workplaces** |
| --- | --- | --- | --- |
| Y=Yes  N=No |  |  |  |
| S=Somewhat |  |  |  |
| Are control strategies associated with modes of transmission? | S^1^ | S | S^2^ |
| Are multiple controls required/recommended? | Y | Y | Y |
| Are control strategies based on risk level? | N | N | S^3^ |
| Are control strategies based on the hierarchy of controls? | N | N | N |
| Does the standard mention source control? | Y^4^ | Y^4^ | Y^4^ |
| Does the regulation address these specific controls? |  |  |  |
| Screening workers | N | N | Y^5^ |
| Vaccination | N | N | N |
| Testing | N | N | Y |
| Physical distancing | Y | Y | Y |
| Limit occupancy numbers | N | N | N |
| Cleaning and disinfection of surfaces and objects | Y | Y | Y |
| Hand washing | Y | Y | Y |
| Engineering controls | S^6^ | S^6^ | Y^6^ |
| Ventilation | N | N | Y |
| Ventilation filtration | N | N | Y |
| Increase outside air | N | N | Y |
| Portable HEPA filtration units or other air cleaning devices | N | N | Y |
| Administrative controls | S | S | Y^7^ |
| Employee training | N | N | Y |
| Case investigation/contract tracing | N | N | Y |
| Policy or procedure for reporting exposure | N | N | Y |
| Recordkeeping | N | N | Y |
| Exclusion of cases/medical removal | Y | Y | Y |
| Access to testing at no cost to employee | N | N | Y |
| Return to work criteria | N | N | Y |
| Access to medical records | N | N | Y |
| Privacy of medical records | N | N | N |
| Personal protective equipment | S | S | Y |
| Use of face coverings | S^8^ | S^8^ | Y^9^ |
| Use of face shields | N | S | Y |
| Use of barrier shields | Y | Y | Y^10^ |
| Gloves | N | N | Y^11^ |
| Goggles | N | N | Y^12^ |
| Use of facemasks (surgical) | N | N | Y^13^ |
| Use of respirators | N | N | Y^14^ |
| Voluntary use of respirators | N | N | Y |

Notes: Adapted from Brosseau et al. 2023 and applied to Oregon ETS and Governor’s Executive Order regarding farm labor housing, work sites and transportation.

1. “ Clean the facilities and equipment before each occupancy. Ensure that high-touch areas in common use facilities are sanitized at least two times daily (if housing occupants are assigned this task, it must be treated as a work assignment rather than a voluntary action”

2.”Employers must clean and disinfect any common areas, high-touch-surfaces, and any shared equipment under the employer’s control that an individual known to be infected with

COVID-19 used or had direct physical contact with. For exceptional risk workplaces, training is required that includes ‘An explanation of contact, droplet, and airborne modes of

transmission of COVID-19, including…how employees can take precautionary measures to minimize their exposure.”

3. General workplaces must consider physical distancing and occupancy levels.

4. Source control means the use of protective equipment or other measures such as face coverings to prevent the spread of illness from a potentially infectious person to others.

5. Worker screening in personal services, construction, K-12 institutions, higher education, emergency medical services; student screening in K-12 institutions, higher education; screening of all individuals in healthcare. Screening includes asking about symptoms of COVID-19; instructing employees to self-monitor for symptoms consistent with COVID-19; directing workers/students to conduct a self-check for COVID-19 symptoms before coming to work/campus; asking if workers/clients have been advised to self-quarantine because of exposure to someone with COVID-19 or if they have been told to isolate after testing positive for COVID-19.

6. Engineering controls include erecting barriers between workers, methods for separating people by 6 ft, ventilation system improvements

7. Use of administrative controls as a means of limiting exposure, including employee training, changing scheduling and how employees conduct their work, recordkeeping, and return to work criteria (contact tracing or case investigation and procedures)

8. Face covers/cloth facial covering…but doesn’t specific that employers MUST provide at no cost to employees

9. Provide masks, face coverings, or face shields for employees at no cost to the worker; When employees are transported in a vehicle for work purposes, regardless of the travel distance or duration

involved, all occupants in the vehicle must wear a mask, face covering, or face shield

10. As part of assessment of risk (e.g., “How have engineering controls such as ventilation (whether portable air filtration units equipped with HEPA filters,

airborne infection isolation rooms, local exhaust ventilation, or general building HVAC systems) and physical barriers been used to minimize employee exposure to COVID-19?”), required for Workplaces at Exceptional Risk (e.g, “Use physical barriers or partitions in triage areas to guide patients when appropriate”) and as option for bars, restaurants, retail stores, etc.

11. Healthcare: Whenever an employee provides direct patient care for a patient known or suspected to be infected with COVID-19, the employer must provide the affected worker with

gloves, a gown, eye protection (goggles or face shield), and a medical-grade mask or a NIOSH-approved respirator. Glove use also described for personal service providers, childcare and

early education, higher education, emergency medical services. [ETS and PS: Veterinary services and EMS have specific requirements for gloves.]

12. Goggle use described for emergency medical services, healthcare.

13. Emergency Medical Services only.

14. Respirator use described for emergency medical services, healthcare.
