# Supplementary material for: Emergency temporary standards and COVID-19 trends among Oregon farmworkers
Source: PLoS One. 2025 Aug 8;20(8):e0329130. doi: 10.1371/journal.pone.0329130 (PMC12334050; doi:10.1371/journal.pone.0329130)
Supplement: S1 Text — (DOCX) [file pone.0329130.s004.docx]

S1 Text. Interpolating general population and number of agricultural in Oregon

We interpolated the number of agricultural workers for our study period using data from the 2017 and 2022 Census of Agriculture.  For each county we assumed each type of worker category changed linearly, i.e., for each county a slope and y-intercept were calculated based on the number of persons in each category and the dates in which the 2017 and 2022 censuses were conducted.  Phase 3, which was the phase focused on  data collection, of the U.S. Agricultural Census ran from November 2022 – May 2023. The middle point of these 7 months is February 14th. Hence February 14th 2018 and February 14, 2023 were selected as the dates in which these censuses happened. The number of workers in each category for each day between March 1st, 2020 and March 31st, 2021 were calculated by inserting the day number in the linear model calculated for each county.  A similar method was employed to estimate the total population in each county. In this case the population estimates from April 1, 2020 to July 1, 2021 from the American Community Survey were used to build the linear models that provided the population estimates for each county by linear inter and extrapolation. As in the case of (Lusk and Chandra, 2021), we assumed that for each category the number of agricultural workers with COVID-19 is equal to their proportion of the total population.
